# GLAD-Derived Silicon Nanoarrays on Electrochemically Polished Cu Foil: A Promising Anode for High-Performance Lithium-Ion Batteries

**DOI:** 10.1021/acsami.5c05422

**Published:** 2025-06-13

**Authors:** Sourav Mallick, Xiaosong Huang, Ram B. Gupta, Dexian Ye

**Affiliations:** † Department of Chemical and Life Science Engineering, 6889 Virginia Commonwealth University, Richmond, Virginia 23219, United States; ‡ Materials & Manufacturing Systems Research Laboratory, General Motors Research & Development Center, Warren, Michigan 48090, United States; § Department of Physics, 6889 Virginia Commonwealth University, Richmond, Virginia 23284, United States

**Keywords:** GLAD, electrochemical polishing, silicon, nanospring, lithium ion battery

## Abstract

Nanostructured silicon (Si) anodes with various dimensions (0- or 1D) are widely explored in the manufacturing of high-energy-density lithium-ion batteries (LIBs) to mitigate volume expansion during cycling. However, most of them suffer from multiple issues, such as phase impurity, inhomogeneity in particle size, and poor mechanical strength, resulting in poor rate capability, cycle performance, and Coulombic efficiency. In this work, a modified physical vapor deposition technique, known as the glancing angle deposition (GLAD) method, is utilized to produce pure Si nanospring arrays on Cu foil. The nanospring architecture, with controlled dimensions, facilitates Li^+^ diffusion throughout the amorphous Si and offers good rate performance. In this case, electrochemical polishing of Cu foil has played a pivotal role to achieve a very high specific capacity of 2800 mAh g^–1^ at 300 mA g^–1^ and a very good rate capability of up to 4500 mA g^–1^. The electrochemical polishing facilitates uniform deposition of the Si nanosprings on the Cu surface and offers better structural robustness compared to the unpolished one.

## Graphical abstract

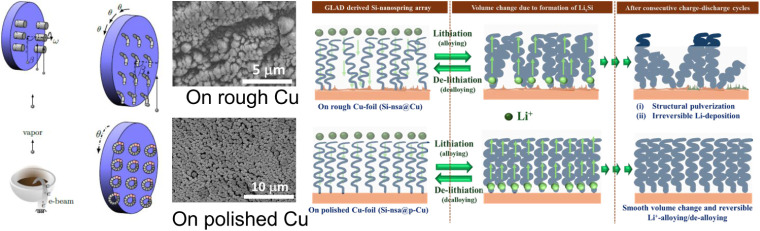

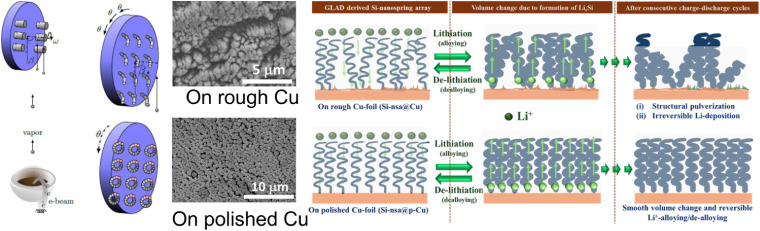

## Introduction

Increasing production of electric vehicles (EVs) demands efficient Lithium-ion batteries (LIBs) with high energy density and specific capacity. The performance of LIBs is largely dependent on the electrochemical performance of the cathode and anode materials. While there are various kinds of cathode materials, including LiCoO_2_ (LCO), LiNi_*x*_Co_*y*_Mn_*z*_O_2_ (NCM), LiMn_2_O_4_ (LMO), LiNi_*x*_Co_*y*_Al_*z*_O_2_ (LCA), LiFePO_4_ (LFP), etc., that are well explored,^1^ only a few types of anode materials, such as graphite, silicon (Si), and Li_4_Ti_5_O_12_ (LTO), are utilized for commercial LIB production to date. Among them, the graphite-based LIB anodes are advantageous in terms of tunable band gap, high thermal and electrical conductivity, etc.^2–4^ However, the lower theoretical capacity of 372 mAh g^–1^ (for LiC_6_) of the commercial-grade graphite anodes limits the electrochemical performance of LIBs, which is required for EVs with a long driving range.^4^

Si as a replacement for graphite anode material in LIBs has been researched for decades. Si has a theoretical gravimetric capacity of 3579 mAh g^–1^ and a volumetric capacity of 8303 mAh cm^–3^ (for Li_15_Si_4_ at room temperature).^5,6^ Therefore, a pure Si anode can provide about 10 times the capacity of current graphite anode-based LIBs. Nevertheless, Si anodes in LIBs suffer from a quick loss of energy capacity. The degradation of the electrochemical performance of active materials (both Si active sites and Li ions) during charge/discharge cycles occurs due to significant volume changes.^7^ Si anodes suffer from severe volumetric expansion during lithiation, leading to poor cycling stability. The volume variation can be ∼300% during lithiation–delithiation cycles.^8–10^ Such large volume variation causes the gradual pulverization and delamination of the anode material, resulting in the formation of unstable solid-electrolyte interfaces (SEIs) and poor electrical conductivity.^6,8,11–13^ Furthermore, large volume expansion introduces high stress inside anodes, particularly in a direction normal to the current collector, which is usually copper (Cu) foil.^14–16^ SiO_2_-based anodes also suffer from poor electrical conductivity and sluggish electronic charge transfer kinetics.^17^

It is observed that the fracture strength as well as the strain energy release rate of Si particles increase when the size is reduced to micrometer or nanometer scales.^18^ There is a critical size of Si (∼150 nm) at which the fracture strength is high enough to sustain the stress introduced by lithiation.^19^ This has led to extensive research on a large variety of Si nanoarchitectures as the active anode material in LIBs, including Si nanoparticles, Si nanospheres, Si/graphite core–shell nanoparticles, Si nanowires, Si nanorods, Si nanotubes, Si nanosheets, Si thin films, encapsulated Si nanoparticles (“yolk–shell” or “pomegranate” like structures), mesoporous Si nanostructures, and hierarchical Si nanostructures.^20–31^ Among all the Si nanostructures studied, Si nanoparticles and their composites are the most investigated category due to their crack-resistant properties and relatively easy preparation method. Although the performance of small Si nanoparticles (∼10 nm in diameter) seems promising, they are prone to aggregation and coalescence without being anchored in a matrix of another material to form composite anodes. The diluted nanoparticles in the matrix significantly reduce the mass loading of the active material for LIBs. Si nanoparticles have other drawbacks for application in LIB electrodes: (1) the immense surface area consumes Li ions through the formation of unstable SEI, hence limiting the Coulombic efficiency; and (2) the electrical contacts between particles, as well as with the current collector, are weak.^32^ As such, the resistivity is high, and the thickness of the anode is limited. Moreover, the Si nanoparticles derived from traditionally used high-energy mechanical milling processes suffer from issues such as phase impurity and inhomogeneity in particle size distribution. Other types of Si nanostructures are extensively investigated, including Si nanowires, Si nanofibers, Si nanotubes, porous Si microparticles, and Si nanoparticle assemblies. One-dimensional (1D) nanostructures (i.e., nanowires, nanofibers, nanotubes) can be directly grown on a current collector, thus providing stronger mechanical contact and better electrical conductivity than those of nanoparticles. However, volumetric expansion is anisotropic in 1D nanostructures due to the rate-controlling lithiation mechanism. Individual nanostructures undergo anisotropic swelling, neckling, and eventually buckling during the lithiation/delithiation cycles, as revealed by in situ transmission electron microscopy experiments.^33–36^ The stability and electrical conductivity of 1D Si nanostructure-based active anode materials can be improved by coating them with carbon or silicon oxide layers. However, the cost of processing is high, and the mass loading is low for practical applications, particularly for EVs.^37^ Porous Si microspheres and other types of porous Si films demonstrate excellent behavior in accommodating volume expansion during the lithiation process. However, their mechanical strength is poor, and the volumetric energy density is low.

The idea of using flexible Si nanosprings as the anode material in LIBs has been tested preliminarily by Chasiotis’s group.^38^ The in situ testing of individual Si nanosprings inside a scanning electron microscope (SEM) reveals that they can accommodate significant volumetric expansion when alloying with Li, thus sustaining the stress induced by lithiation.^38^ Their results also indicate that the fibrillar morphology of the Si nanosprings enhances the diffusivity of Li^+^ in amorphous Si-based anodes.^38^ The unique fibrillar feature is formed by the physical-vapor-deposition-based glancing angle deposition (GLAD) technique. Polat et al. also fabricated Si–Cu composite nanosprings with ∼10% Cu on Cu discs by using GLAD.^39,40^ The electrochemical testing of Si–Cu composite nanosprings shows a remarkably high initial capacity of 3130 mAh g^–1^ and good capacity retention over 100 cycles.^39^ Similar Si–Cu composite anodes have been synthesized by Wang et al. using GLAD and thermal annealing.^41^ However, the mass loading of their Si–Cu composite nanospring anode is low, as the thickness of their samples is less than 1 μm in both cases, and the rate as well as the cycling performances are comparatively low. The novelty of our work lies in the fact that the GLAD-derived Si nanospring array achieved a very good specific capacity as well as rate capability while the electrochemically polished Cu foil is utilized as the substrate (in this case, the current collector), and it outperforms many of the recently reported Si-based anodes. Moreover, no significant structural pulverization is observed after consecutive charge–discharge cycles at a high current density of 2100 mA g^–1^.

## Results and Discussion

### Glancing Angle Deposition (GLAD) Technique

GLAD is a mature technique for fabricating nanospring arrays based on widely used physical vapor deposition (PVD) systems in the industry. The advantages of GLAD in preparing nanospring arrays include its room-temperature process, lack of limitations regarding material and substrate types, and ease of control.^42–44^ In GLAD, the source material is evaporated by a common PVD method. The substrate is mounted on a surface to allow the vapor of the source to approach the substrate at a large incident angle β (usually β > 75°) with respect to the substrate normal. During the deposition process in GLAD, the substrate is rotated in the surface normal direction by a stepper motor, as depicted in Figure 1a. The large incident angle introduces a shadowing effect on the growing interface, limiting the protruded parts of the surface from continuing their growth.^44^ As the substrate is maintained at a relatively low temperature, the deposited atoms exhibit low diffusivity. Consequently, nanowires are formed spontaneously, well-aligned in a direction toward the PVD source.

**Figure 1:**
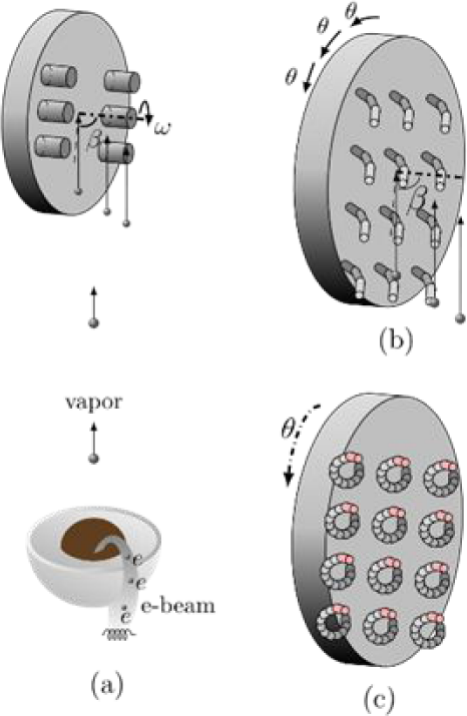
Glancing angle deposition (GLAD) technique based on an e-beam evaporator system and the principle of the growth of nanosprings in GLAD. (a) E-beam evaporated vapor approaches a rotating substrate with a large incident angle β and hence the well-aligned nanorods form due to shadowing effect. (b) Curved nanowires are formed when the substrate is held in position for a short period of time and abruptly rotated by a small azimuthal angle θ with a large incident angle β. (c) When the “stop-and-go” motion pattern continues, round nanosprings can be fabricated in GLAD.

### Electrochemical Polishing of Cu Foil

The topology of substrates is crucial in fabricating uniform nanostructures using the GLAD method. Any nodular defects on the surface will amplify the shadowing effect, thus preventing the growth of nanostructures in a wide vicinity around the defects. In particular, when the substrate is rotated, isolated microstructures will be generated on those nodular defects.^45^ Atomically flat and smooth surfaces or substrates containing prefabricated regular patterns are used to grow uniform nanostructures in GLAD. On the other hand, the morphology of Cu foils used in LIBs as the current collectors contains irregular ridges and grooves. Therefore, it is necessary to polish the Cu foil surface before the fabrication of nanosprings by using the GLAD method. Hence, the Cu foils used in this study were first electropolished using a phosphoric acid–based electrolyte in a water-cooled 1000 mL electropolishing cell (Kristall 620, 500 mL of 75% phosphoric acid, 250 mL of DI water, and 250 mL methanol). The SEM images of electrochemically polished and unpolished Cu foils are shown in Figure 2. Comparing the SEM images, it can be concluded that the electropolishing technique has effectively removed the irregularities of the Cu surface to a large extent.

**Figure 2:**
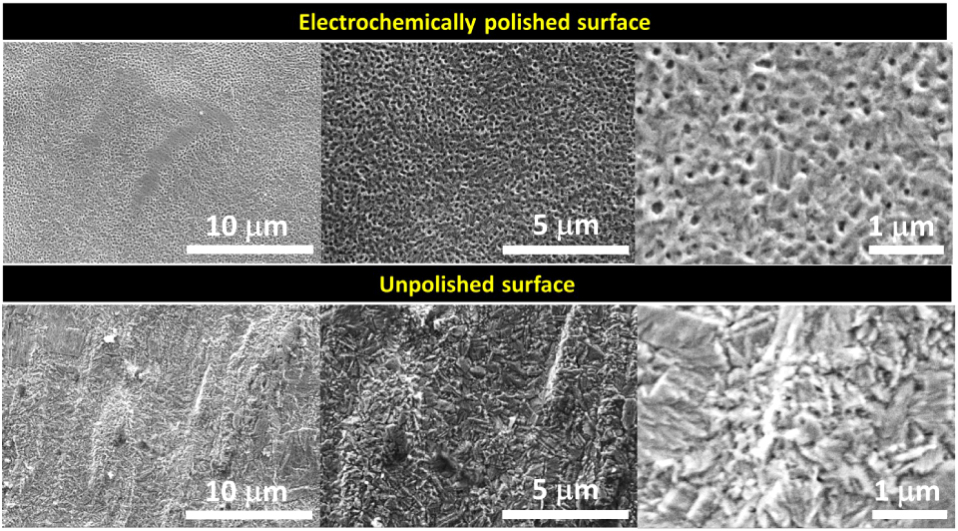
SEM images of the surface of electrochemically polished and unpolished Cu foil.

### Deposition of Si Nanospring Arrays

In GLAD, spring-shaped nanowires are formed with slow substrate rotation. Therefore, a “stop-and-go” substrate rotation strategy is designed to fabricate Si nanospring arrays on Cu foils. First, the substrates are held still for the deposition of inclined nanowires at β = 85°. Once the desired length of the nanowires is reached, the substrate is abruptly rotated by an azimuthal angle θ (on the plane of the substrate surface) to continue the growth of the second segment of the nanowires. The growth direction of the second nanowires also turns by the same azimuthal angle θ, as shown in Figure 1b. After *n* = 360/θ steps, one turn of the spring is completed, as demonstrated in Figure 1c. We have demonstrated success in growing Si nanosprings on Cu foils by the GLAD technique. A pure Si source is evaporated from an e-beam evaporator with a 10 kW power supply. The distance between the source and substrate is about 50 cm. The e-beam source has four pockets to evaporate different materials. The Cu foil substrate is mounted on a stainless-steel plate attached to a stepper motor. A custom-built goniometer is used to tilt the substrate and set the incident angles for GLAD. The angle can be changed manually from outside without breaking the vacuum. A computer-controlled stepper motor drives the rotation of the substrate during deposition. In the fabrication of nanosprings, 25 motions cover one revolution of the rotation. Therefore, each motion sweeps an angle θ ≅ 15°. After each move, the substrate is held at its current position for a period of time. In our experiment, the Si deposition rate is about 0.1 nm/s. The holding time is thus set to 20 min. Once a full turn of the Si nanospring is completed, the source is changed to nickel (Ni) for surface coating of the nanospring. The incident angle β is changed to 45° for better coverage of the coating on the surface of Si nanosprings. The thickness of the Ni coating is about 10 nm, measured using a quartz crystal microbalance. Ni coating improves the electrical conductivity of the Si nanosprings, while it is electrochemically inactive to Li^+^.^46^ The amount of Ni coating is about 1% of the total Si loading in the anode by weight. A total of five turns of nanosprings are fabricated in this study. The Si nanospring arrays deposited on polished and unpolished Cu foil are abbreviated as Si-nsa@p-Cu and Si-nsa@Cu, respectively, throughout the manuscript.

### SEM Analysis of Si Nanospring Arrays

The SEM images of the Si nanosprings grown on the polished Cu foil surface are shown in Figure 3 and on the unpolished Cu foil are included in Figure S1. Similar fabrication parameters were applied to produce the four turns of Si nanospring arrays on both polished and unpolished Cu foil surfaces. The width and the gap between the Si stripes are calculated by using the top-view images of the anode surface using ImageJ software. Comparing the top-view SEM images of Si-nsa@p-Cu (Figure 3a) and Si-nsa@Cu (Figure S1a), it is observed that the nanosprings are uniformly grown on the polished Cu foil surface without stripes and gaps, whereas, in the case of the unpolished Cu foil, the nanosprings are not smooth, and large gaps of 1.4 ± 0.2 mm between Si stripes of 3.8±0.8⁡μm are visible. The high-magnification SEM top-view image of the Si nanosprings grown on the polished Cu foil surface shows the fibril structure, as shown in Figure 3b. The width of individual Si fibers in the nanospring is 11 ± 3 nm. The helix angles can be measured from the cross-sectional SEM images of nanosprings grown on the Si/PMMA surface. In order to prepare the cross-section of the sample, a piece of Si substrate coated with poly­(methyl methacrylate) (PMMA) was placed on the side of the Cu foil during the growth. From the cross-sectional image shown in Figure 3c, the helix angle of the springs is 43 ± 5° with respect to the substrate surface. The height and diameter of each of the Si nanosprings are measured as 2.71 ± 0.04 μm and 329.8 ± 48.9 nm, respectively, using ImageJ software on Figure 3c. The GLAD parameters and characteristics of the Si nanoarrays are summarized in Table 1.

**Table 1:** GLAD Parameters and the Dimensions of Si Nanospring

GLAD parameters				Dimension of Si nanosprings		
Power of e-beam	Distance (source-substrate)	Si deposition rate	Holding time	Diameter (nm)	Height (μm)	Helix angle (°)
10 kW	50 cm	0.1 nm s^–1^	20 min	329.8 ± 48.9	2.71 ± 0.04	43 ± 5

**Figure 3:**
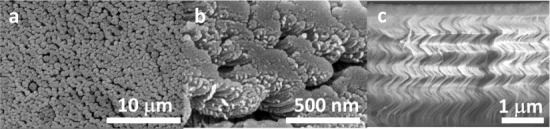
SEM images of four-turn Si nanosprings grown on polished Cu foils: (a) top-view image of Si nanosprings, (b) high magnification of Si springs on polished Cu foil, and (c) cross-section image of Si nanosprings on the Si/PMMA surface.

### Effect of Polishing on Electrochemical Performance

The electrochemical performance of both the Si-nsa@Cu and Si-nsa@p-Cu electrodes was examined in a half-cell configuration with respect to Li foil. Before the assembly of the Li-ion battery half-cells, approximately 1% of binders consisting of poly­(acrylic acid) (PAA) and carboxymethyl cellulose (CMC) (PAA/CMC = 50/50 wt/wt) were spin-coated onto the surface of the Si nanosprings. The binders were cured in a quartz tube furnace under vacuum at 150 °C for 60 min. The coin cells (CR2302) were assembled using 1.0 M LiPF_6_ in ethylene carbonate/ethyl methyl carbonate (EC/EMC = 50/50 (v/v)) as the electrolyte. It is interesting to note that a significant improvement in electrochemical performance (Figure 4) is observed in the case of polished Cu foil (Si-nsa@p-Cu electrode). Figure 4a shows the charge–discharge profiles of both samples at 300 mA g^–1^. The Si-nsa@p-Cu electrode achieves a specific capacity of 2840 mAh g^–1^, which is almost 2.3 times higher compared to the Si-nsa@Cu electrode (1209 mAh g^–1^). The Si-nsa@p-Cu electrode shows a very high specific capacity within a wider window of current density, ranging from 300 to 4500 mA g^–1^. Second charge–discharge cycles at each current density are shown in Figure S2. Figure 4b shows the superiority of Si-nsa@p-Cu electrode compared to the Si-nsa@Cu electrode in terms of rate capability. It shows specific capacities of 2840, 2640, 2508, 2471, 2297, 2123, 2011, 1924, 1849, 1713, 1623, 1561, 1412, 1325, and 1300 mAh g^–1^ at current densities of 300, 600, 900, 1200, 1500, 1800, 2100, 2400, 2700, 3000, 3300, 3600, 4000, 4200, and 4500 mA g^–1^, respectively. 94% of the initial value was achieved once the current rate was decreased to 300 mA g^–1^ again, signifying good retention of electrochemical performance. On the other hand, the Si-nsa@Cu electrode shows specific capacities of 1209 and 920 mAhg^–1^ at the current densities of 300 and 1200 mA g^–1^, respectively (Figure 4b and S3). Charge storage in the Si nanospring anode follows the usual alloying–dealloying mechanism.^7,47^ As a control experiment, the electrochemical performance of Si-nsa@Cu was evaluated without using any binder, and it shows very poor electrochemical performance in the absence of binders (Figure S4). The charge storage performance of the Si-based electrode largely varies with the applied current density due to the varying Li^+^ diffusion behavior as well as the different extent of volume change during the dealloying/alloying process upon a consecutive charge–discharge process.^48–50^ Hence, the cycling performance of the Si nanospring-based anode was examined at both lower and higher current densities. The cycling performance of Si-nsa@Cu and Si-nsa@p-Cu electrodes for 100 cycles at 600 mA g^–1^ is shown in Figure 4c. The Si-nsa@p-Cu electrode shows retentions of 91% and 59.4% in specific capacity after 50 and 100 cycles, respectively, whereas the Si-nsa@Cu electrode shows comparatively poor retention of 82% and 54% after 50 and 100 cycles, respectively. The importance of polishing the Cu foil is further realized from the impedance analysis of both the electrodes after 50 charge–discharge cycles (Figure 4d). Si-nsa@p-Cu shows a charge-transfer resistance (*R*_ct_) of 54.86 Ω, which is significantly lower compared to that of the unpolished one (73 Ω). It is quite striking to note that, although there is not much difference between the morphological features of GLAD-derived Si nanospring arrays grown on polished and unpolished Cu foil, their electrochemical performance varies significantly. Hence, it can be obviously postulated that electrochemical polishing plays a critical role in improving performance. From SEM analysis (Figures 2 and 3), it is already proven that the polished Cu surface is free from most of the irregular edges/grooves and ensures a uniform deposition of Si nanoarrays through GLAD (Si-nsa@p-Cu). As illustrated in Figure 4e, in this case, each of the Si nanospring units behaves almost identically during a consecutive charge–discharge process and experiences a similar extent of volume change upon alloying/dealloying with Li^+^. This further ensures long cycling and good rate performance of the as-synthesized pure Si-based anode without much structural pulverization. This is proven by the postcycling SEM of Si-nsa@p-Cu (Figure S5), which shows that there is not much cracking on the surface of the anode after cycling. On the other hand, the rough surface of the unpolished Cu foil hinders the uniform deposition of Si nanosprings (Si-nsa@Cu), which further aggravates the severity of the volume expansion of Si upon alloying with Li^+^ and promotes structural damage to the anode. A greater extent of irreversibility in alloying/dealloying during electrochemical potential-induced cycling may also lead to electrolyte decomposition (Figure 4e).

**Figure 4:**
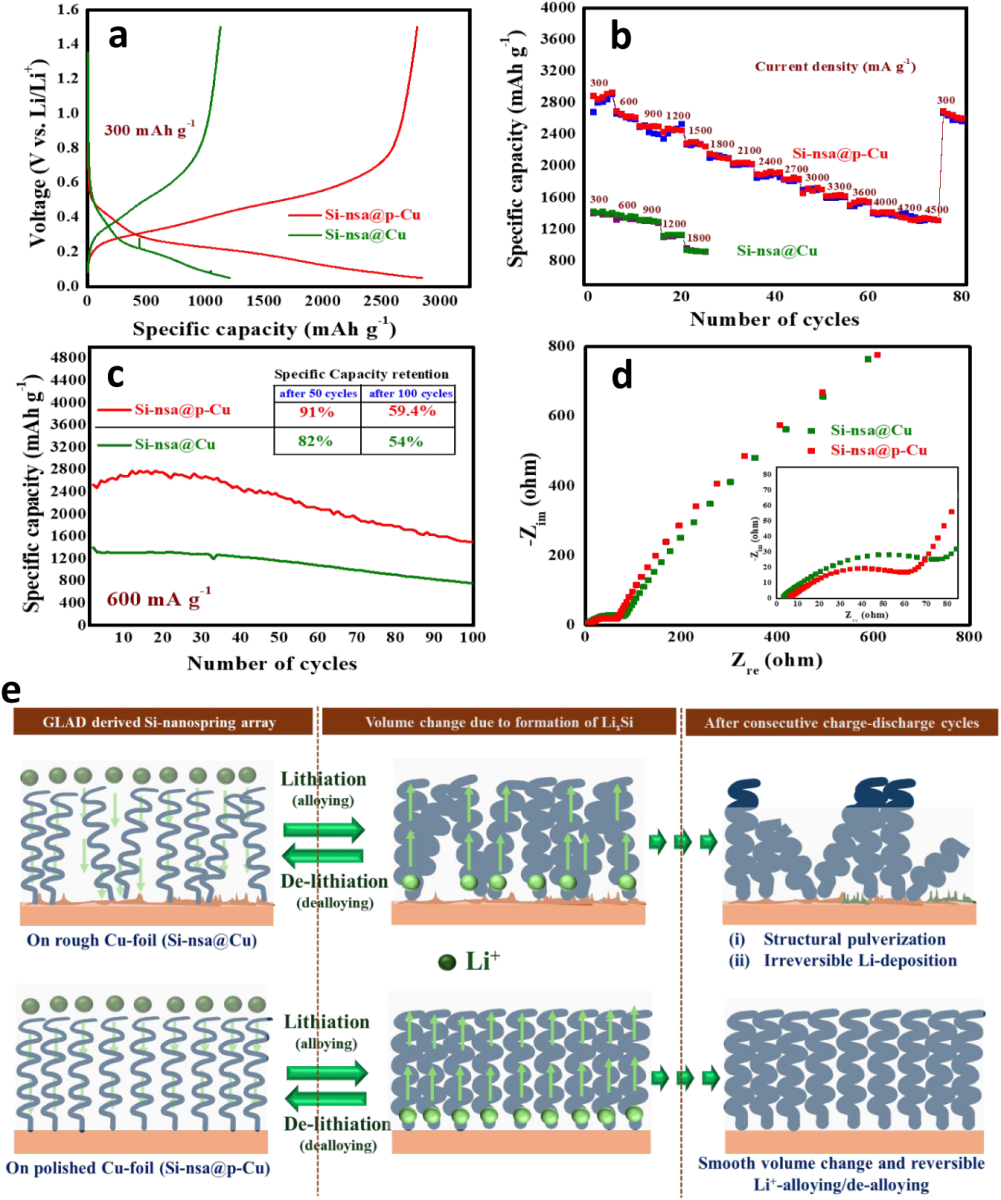
Comparison between Si-nsa@Cu and Si-nsa@p-Cu in terms of (a) galvanostatic charge–discharge performance at 300 mA g^–1^, (b) rate capability plot, (c) cycling performance at 600 mA g^–1^, and (d) impedance behavior. (e) Schematic representation of the effect of polishing of Cu foil on the electrochemical performance of the Si anode.

The effect of cycling on the Si anode is further evaluated by differential capacity (dQ/dV) analysis at various stages of cycling (Figure 5a). All of the dQ/dV profiles are composed of two pairs of peaks corresponding to the alloying and dealloying processes during the discharge and charge of the Si anode within the voltage window of 0.04–1.5 V vs Li/Li^+^.^51^ The discharge peaks, observed at 0.25–0.29 V corresponding to the transformation of amorphous Si (a-Si) to Li_2.0_Si followed by the next alloying step at 0.09–0.1 V, which is attributed to the transformation of a-Li_2.0_Si to a-Li_3.5_Si. The charging process follows the reverse transformation, including Li_3.5_Si to Li_2.0_Si and Li_2.0_Si to Si at 0.25 and 0.45 V vs Li/Li^+^, respectively. Upon cycling, the intensity of all the redox peaks decreases, indicating decreased capacity. The extent of the peak shift and decrease in intensity becomes more significant after 100 cycles. Hence, it is proven that the GLAD-derived nanospring arrays can efficiently withstand the volume change during the alloying/dealloying process to a large extent (Figure 4c) and offer a good cycling performance. In order to understand the effect of cycling in detail on Si nanosprings, electrochemical impedance analysis was performed at various stages of cycling at 600 mA g^–1^. The fitted Nyquist plots are shown in Figure 5b, and the equivalent circuit is shown in Figure S6. Here, it is observed that the charge transfer resistance (*R*_ct_), indicated by the depressed semicircle in the high-frequency region of the Nyquist plot, increases with the progress of cycling. It is worth noting that the *R*_ct_ changes from 51.92 to 54.86 Ω after the 50th cycle and further increases to 95 Ω at the end of the 100th cycle. This implies that the degree of volume change during the alloying/dealloying process of the as-synthesized Si nanospring-based anode is less severe during the first 50 cycles, leading to a lesser extent of performance deterioration initially. Figure 5c shows the cycling performance of the Si-nsa@p-Cu electrode at a very high current density of 2100 mA g^–1^. In this case, the anode shows a specific capacity of 1344 mAh g^–1^ after 100 cycles and further decreases to 795 mAh g^–1^ after 200 cycles. The initial Coulombic efficiency at this higher current density is 54%, which abruptly improves to 99% from the second cycle and is retained up to 200 cycles. Although commercial graphite anodes show promising cycling performance (over 1000 cycles) at a lower current density of 100 mA g^–1^, they face several issues like uneven SEI formation, capacity fading, etc., at the higher current density.^52^ On the other hand, although commercial LTO can retain up to 70–75% of capacity after 1000 cycles at high current density, the low theoretical capacity of 175 mAh g^–1^ is the primary bottleneck for its high-energy application.^53^ The electrochemical performance of our GLAD-derived Si nanospring array is compared with recent literature on Si-based anodes of different morphologies and synthesized through different manufacturing routes in Table S1. The as-synthesized Si-nsa@p-Cu anode achieves better rate capability and cycling performance compared to most of the reports published in the last 10 years on silicon-based anodes. It also shows a higher specific capacity compared to commercial graphite and LTO anodes even at higher current density.

**Figure 5:**
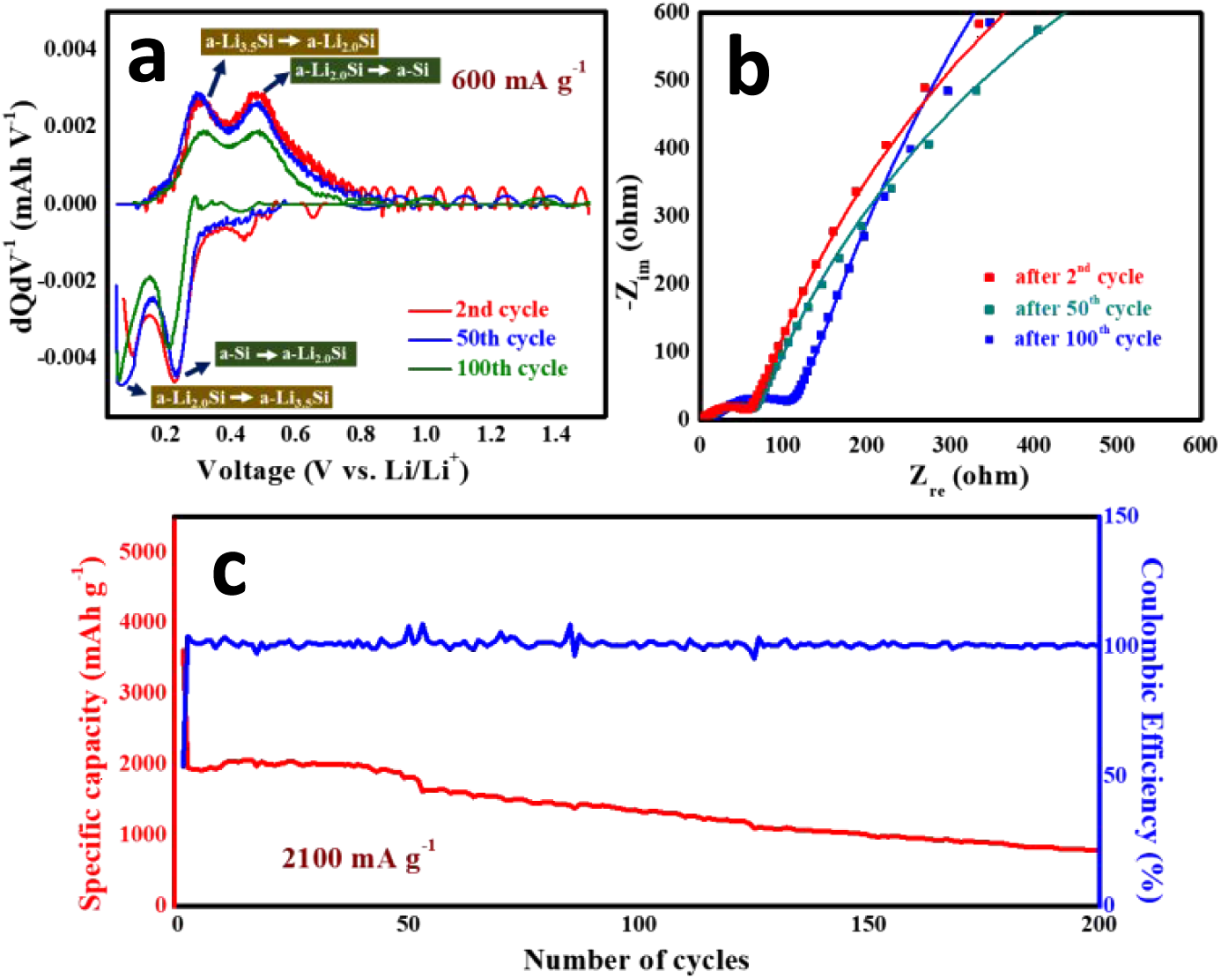
(a) dQ/dV and (b) Nyquist profiles at different stages of cycling at 600 mA g^–1^ of Si-nsa@p-Cu. (c) Cycling performance of Si-nsa@p-Cu at a higher current density of 2100 mA g^–1^.

## Summary

In this study, we fabricated pure Si nanospring arrays on unpolished and electrochemically polished Cu foils with a high Si content by the GLAD method. Here, it is demonstrated that better control over the uniformity of the Si nanospring deposition is achieved by polishing the Cu foils. A significant improvement is observed in terms of the overall dimensions and uniformity of Si nanospring arrays, which plays a critical role in the charge storage performance of Si-based anodes on polished Cu foils. The crucial experimental parameters of the GLAD manufacturing platform are discussed in detail in this article. The as-synthesized Si nanospring-based anodes were tested in the half-cell LIB configuration for the evaluation of electrochemical performance in terms of capacity, rate capability, cycling stability, etc. The capacity of our Si nanospring anode was measured to be 2840 mAh g^–1^ at a current density of 300 mA g^–1^ and retained up to 1300 mAh g^–1^ of specific capacity at the higher current density of 4500 mA g^–1^. Good cycling performance with a specific capacity of 804 mAh g^–1^ at 2100 mA g^–1^ after 200 cycles further proves the benefits of the nanospring architecture of the GLAD-derived Si-based anodes on electrochemically polished Cu foil. The unique morphology of the Si anode successfully mitigates the fast capacity fading related issue caused by severe volume changes during cycling. Electrochemical polishing effectively improves the smoothness of the Cu surface and promotes the even and controlled deposition of the Si nanoarray, which further ensures improved electrochemical performance. This work shows significant improvement in Si-anode manufacturing technology and electrochemical performance. The process is highly reproducible and has great prospect for industrial use in high-energy LIB manufacturing. Further optimization of our electropolishing process and material engineering at the surface/interface of Si nanosprings will be carried out to improve the cycling performance and stability of our anode material.

## Methods

### Materials and Processing

Silicon (lumps, 0.1–2.5 cm, purity >99.9999%) is purchased from Alfa Aesar (now Thermo Scientific Chemicals, Ward Hill, Massachusetts, USA). Nickel (pellets, purity >99.995%) and titanium (pellets, purity >99.995%) are obtained from Kurt J. Lesker Company, Jefferson Hills, PA, USA, without further cleaning or purification. Copper foil (single-side shiny, 25 μm thick, purity >99.8%) is purchased from MTI Corporation, Richmond, CA, USA and used after further electrochemical polishing.

### Cu Foil Polishing

Cu foils are electrolytically polished in a water-cooled 1000 mL agitated electrolytic cell (ATM Kristall 620, Berder Scientific, Germany) using a phosphoric acid-based electrolyte solution (500 mL of 75% phosphoric acid, 250 mL of DI water, and 250 mL of methanol). The polishing time is set to 20 s, and the voltage is 36 V. After polishing, the Cu foils are thoroughly rinsed with running water and then cleaned with DI water.

### Deposition of Si Nanoarray on Polished Cu Foil

Before deposition, a piece of copper foil is cut and cleaned with acetone for 5 min. The foil and silicon substrates are mounted on a substrate holder using a piece of carbon tape. The chamber is evacuated by a mechanical pump and then pumped by a turbo pump to reach a base pressure lower than 5 × 10^–5^ Torr. During the GLAD processing, the background pressure is usually higher than 6 × 10^–6^ Torr. Once the deposition of nanosprings is completed, the samples are removed from the vacuum chamber and spin-coated with the PAA/CMC binders at 1000 rpm for 1 min. The binder is cured under vacuum at 150 °C for 60 min in a quartz tube furnace before assembly of the LIB half-cells.

### Electrochemical Performance Analysis

The electrochemical performance of the GLAD-derived Si nanoarrays was analyzed in the half-cell configuration by coupling them with Li foil. The coin cells (CR2302) were assembled in an argon-filled glovebox using the Celgrade 2340 trilayer microporous membrane as the separator and 1.0 M LiPF_6_ in ethylene carbonate/ethyl methyl carbonate (EC/EMC = 50/50 (v/v)) as the electrolyte. The electrochemical performance is evaluated within the voltage window of 0.04–1.5 V (vs Li/Li^+^). Impedance analysis was performed within the frequency range of 100 kHz to 0.01 Hz at a voltage amplitude of 5 mV. Arbin and MTI battery cyclers were used for the coin cell performance tests.

## Supplementary Material



## Notes

Table of recently reported electrochemical performance of various anode materials in Table S1, with our Si nanospring samples highlighted for comparison; additional charging–discharging performance of the Si nanospring anodes in Figures S2–S4 to demonstrate the necessity of the electropolishing process; additional SEM images of the electrode material are presented in Figures S1 and S4 (PDF)
