# Supplementary material for: GLAD-Derived Silicon Nanoarrays on Electrochemically Polished Cu Foil: A Promising Anode for High-Performance Lithium-Ion Batteries
Source: ACS Appl Mater Interfaces. 2025 Jun 13;17(25):36661–8. doi: 10.1021/acsami.5c05422 (PMC12203465; doi:10.1021/acsami.5c05422)
Supplement: Supplementary file 1 [file am5c05422_si_001.pdf]

Supporting Information

GLAD-derived silicon nanoarrays on electrochemically polished Cu-foil: Promising anode for high-performance Lithium-ion battery

Sourav Mallick[1], Xiaosong Huang[2], Ram B. Gupta[1,*], and Dexian Ye[3,*]

1. Department of Chemical and Life Science Engineering, Virginia Commonwealth University, Richmond, VA 23219, USA
2. Materials & Manufacturing Systems Research Laboratory, General Motors Research & Development Center, Warren, Michigan 48090, USA
3. Department of Physics, Virginia Commonwealth University, Richmond, VA 23284, USA

*Author e-mails: Ram Gupta – rbgupta@vcu.edu; Dexian Ye – dye2@vcu.edu

Figure S1 SEM images of Si-nsa@Cu: (a) Top-view image of Si nanosprings, (b) High magnification of Si springs, (c and d) Cross-section image of Si nanosprings on Si/PMMA surface.

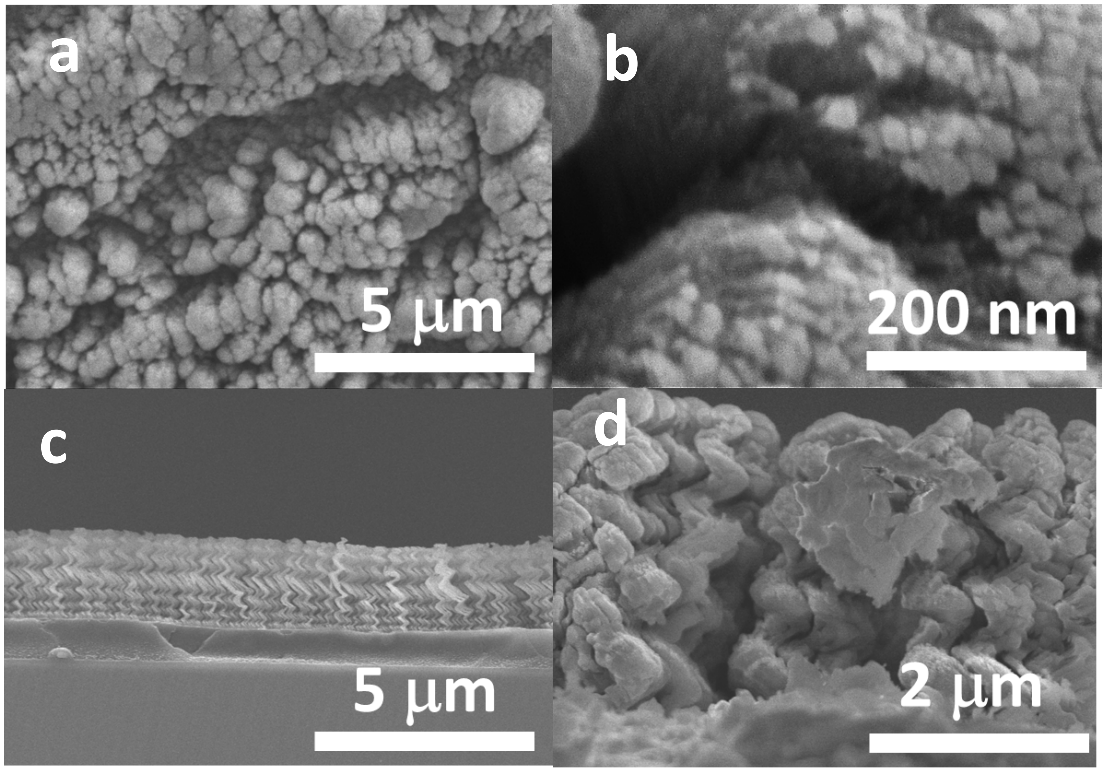

Figure S2 Charge-discharge profiles of Si-nsa@p-Cu at various current density.

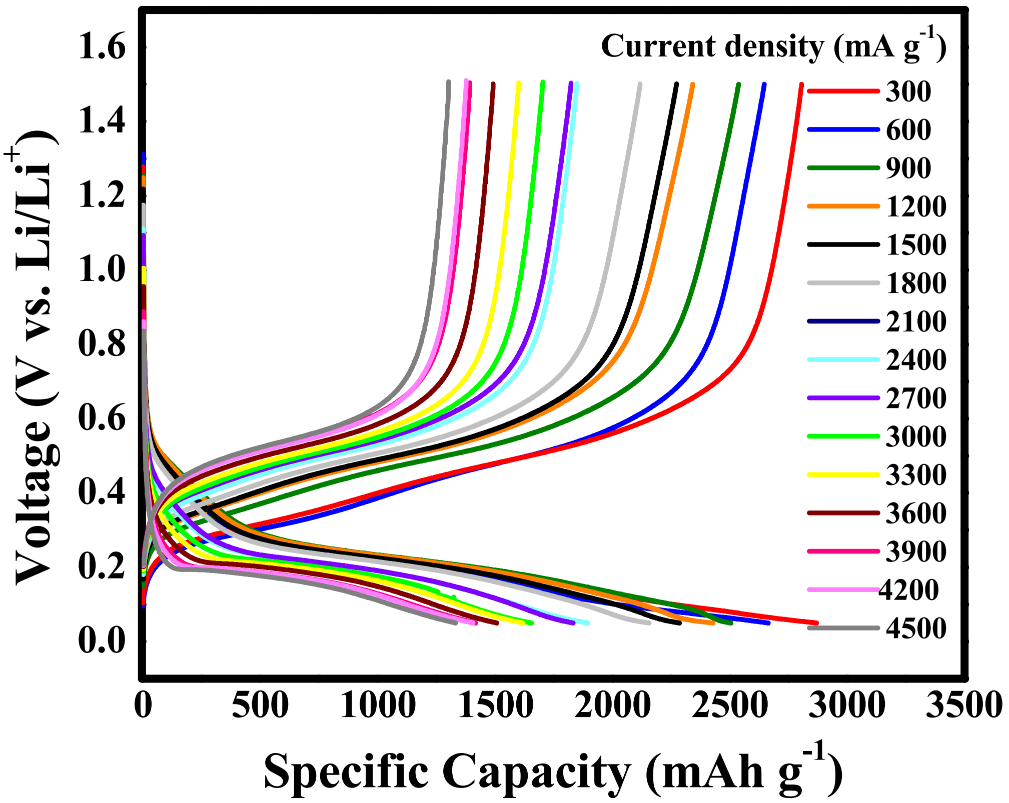

Figure S3 Charge-discharge performance of Si-nsa@Cu at various current density.

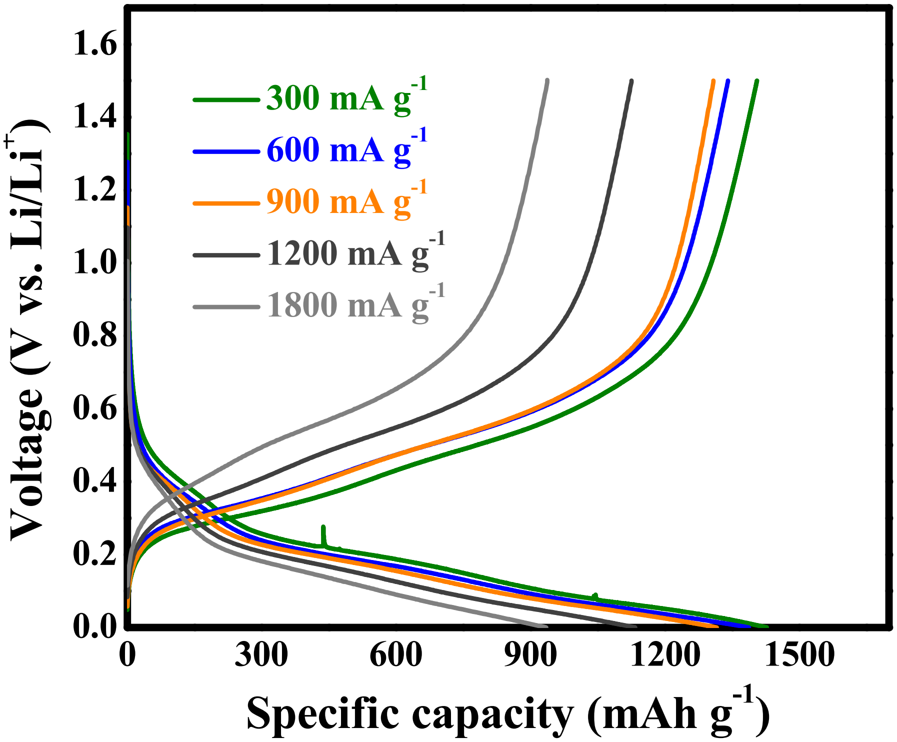

Figure S4 Charge-discharge performance of Si nanospring array on unpolished Cu-foil without binder at various current density.

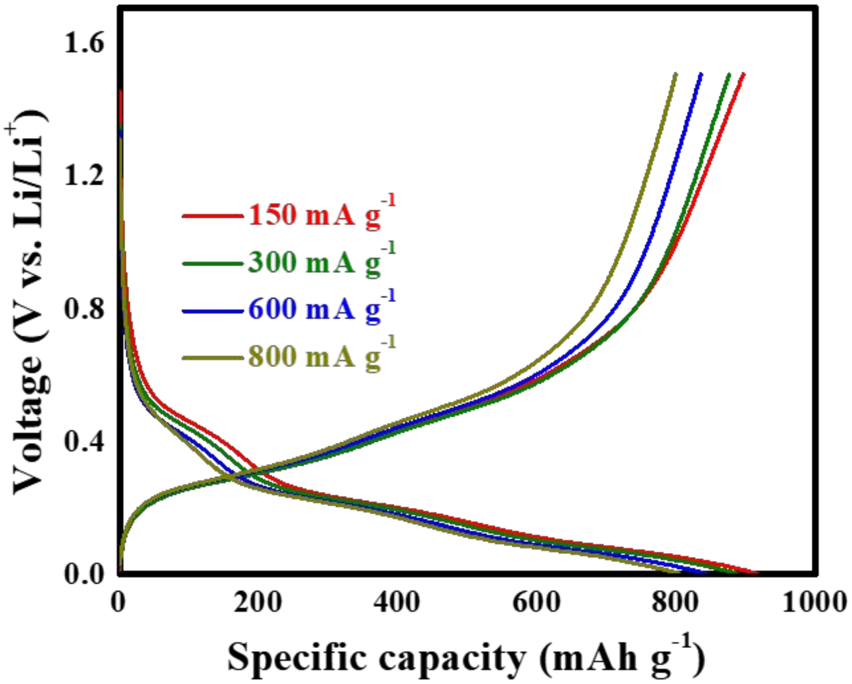

Figure S5 Post-cycling SEM image of Si-nsa@p-Cu at different magnifications.

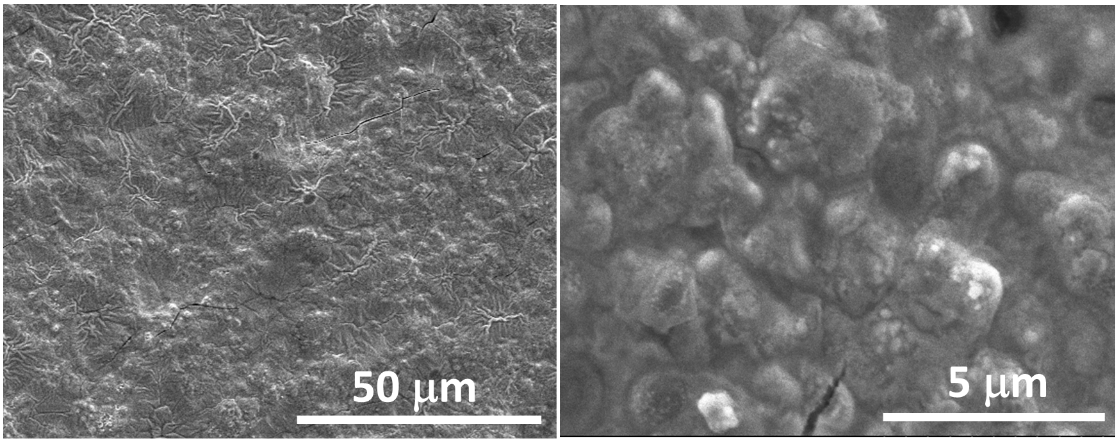

Figure S6 Equivalent circuit for impedance analysis.

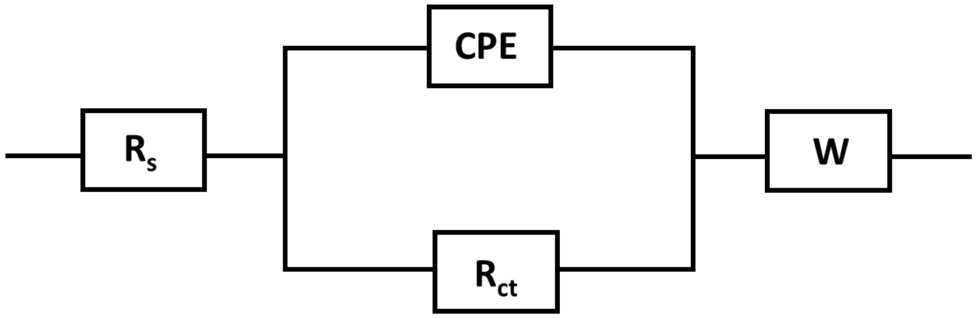

R_s = Solution resistance; R_{ct} = charge-transfer resistance

CPE = Constant phase element; W = Warburg impedance

Table S1 Comparison between the GLAD-derived Si nanoarray on polished Cu-foil with the reported Si-anodes.

Material and morphology	Synthesis process	Rate capability (Sp. Capacity in mAh g^{-1} at a current density in mA g^{-1})		Cycling performance			Ref.
		Highest	Lowest	Specific Capacity ^{1})	Cycle No.	Current density (mAh g^{-} (mA g^{-1})	
Sponge -like Si	Solvothermal at 380 °C	1630 (360)	420 (10000)	1125	100	1000	S1
Porous Si/SiO_x plates	Magnesiothermic reduction	2499 (50)	1443 (1000)	980	100	100	S2
Si-MWCNT nanofilm	PVD	1664 (200)	381 (6400)	1556	100	200	S3
Layered Si	One-step topological reaction	--	1669 (1000)	272.3	20	1000	S4
3D-porous Si	Magnesiothermic reduction and etching	3073 (100)	383 (5000)	2005/ 984	1/200	200	S5
F-functionalized core shell Si-C	High-temperature pyrolysis	694.1 (100)	435 (2000)	683	50	200	S6
Si@C NFs	Coaxial electrospinning	1162.8 (200)	479.7 (2000)	762	100	100	S7
Si@NiAl-LDH	Re-precipitation and *in situ* growth	840.6 (50)	536.3 (5000)	534	60	50	S8
Yolk-Shell SiO_x/C	Sol–gel and selective etching and CVD	1165 (100)	725 (1000)	972	500	500	S9
Si/C	Oxidation, carbon coating followed by acid etching	1362 (200)	743 (2000)	~749.1	300	200	S10
Yolk-shell Si/C	Strong etching	1243 (100)	340 (2000)	1113	200	100	S11
Si@ZIF-glass composite	Melting quenching	1010 (100)	>100 (5000)	650	500	1000	S12
Si-$FeSi_2$-G-C	High energy ball milling	1045 (100)	550 (2000)	925	80	100	S13

Si@Ni-NP/CNTs	Amino functionalization	1200 (100)	746 (1000)	1008	100	100	S14
Carbon coated ant-nest-like Mg-doped microscale porous Si (AMPSi@C)	Multi-step process of thermal nitridation, acid leaching and carbon coating	2134 (420)	1271 (2100)	679	20th to 1000th	2100	S15
Sn-Bonded Si anode (Si–Sn@C400-2)	Multi-step process of slurry coating, carbonization followed by hot pressing	2353 (150)	688.9 (4500)	1009.7	500	1500	S16
Si-core-C-shell	CVD	2200 (100)	----	1892	100	100	S17
Multi-core-Si-void@SiO_2	Heat treatment in air	1282 (100)	-----	1458/1440	200/400	100/1200	S18
Sn-doped SiNWs	Modified alumino-reduction in molten salt	3121 (100)	778 (8000)	1133	500	4000	S19
SiOx@CNTs/C	Low-Temperature Self-Catalysis using ZIF-67 template	1289 (20)	284 (10000)	1080<br>902<br>327	100<br>400<br>1000	500<br>1000<br>5000	S20
Si@CNFs (M1200 Gs)	Modified electrospinning technology	1603.1 (100)	92.3 (5000)	842.3<br>658.1	100<br>100	100<br>1000	S21
**Si-nanospring array on polished Cu-foil (Si-nsa@p-Cu)**	**GLAD-deposition of Si nano-spring on electrochemically polished Cu-foil**	**2840 (300)**	**1300 (4500)**	**2600/ 2325/ 1500**<br><br>**2011/ 1344/ 795**	**2/50/ 100**<br><br>**2/100/ 200**	**600**<br><br>**2100**	**Our work**

References

(S1) Han, Y.; Lin, N.; Xu, T.; Li, T.; Tian, J.; Zhu, Y.; Qian, Y. An Amorphous Si Material with a Sponge-like Structure as an Anode for Li-ion and Na-ion Batteries *Nanoscale* **2018**, *10*, 3153−3158.

(S2) Ruan, H.; Guo, S.; Zhang, L.; Liu, Y.; Li, L.; Huang, Y.; Gao, S.; Tian, Y. Boosting Lithium Storage Performance of Diatomite Derived Si/SiO_x Micronplates via Rationally Regulating the Composition, Morphology and Crystalline Structure *Ceram. Int.* **2022**, *48*, 17510− 17517.

(S3) Wang, Z.; Li, Y.; Huang, S.; Liu, L.; Wang, Y.; Jin, J.; Kong, D.; Zhang, L.; Schmidt, O. G. PVD Customized 2D Porous Amorphous Silicon Nanoflakes Percolated with Carbon Nanotubes for High Areal Capacity Lithium Ion Batteries *J. Mater. Chem. A* **2020**, *8*, 4836−4843.

(S4) Gao, R.; Tang, J.; Terabe, K.; Yu, X.; Sasaki, T.; Hashimoto, A.; Asano, K.; Suzuki, M.-a.; Nakura, K. Preparation of Layered Si Materials as Anode for Lithium-ion Batteries *Chem. Phys. Lett.* **2019**, *730*, 198−205.

(S5) Zuo, X.; Wen, Y.; Qiu, Y.; Cheng, Y.-J.; Yin, S.; Ji, Q.; You, Z.; Zhu, J.; Muller-Buschbaum, P.; Ma, L.; Bruce, P. G.; Xia, Y. Rational Design and Mechanical Understanding of Three-dimensional Macro-/Mesoporous Silicon Lithium-ion Battery Anodes with a Tunable Pore Size and Wall Thickness *ACS Appl. Mater. Interfaces* **2020**, *12*, 43785−43797.

(S6) Chen, X.; Yang, X.; Pan, F.; Zhang, T.; Zhu, X.; Qiu, J.; Li, M.; Mu, Y.; Ming, H. Fluorine-functionalized Core-shell Si@C Anode for a High-energy Lithium-ion Full Battery *J. Alloys Compd.* **2021**, *884*, 160945.

(S7) Zeng, L.; Xi, H.; Liu, X.; Zhang, C. Coaxial Electrospinning Construction Si@C Core−shell Nanofibers for Advanced Flexible Lithium-ion Batteries *Nanomaterials* **2021**, *11*, 3454.

(S8) Li, Q.; Wang, Y.; Lu, B.; Yu, J.; Yuan, M.; Tan, Q.; Zhong, Z.; Su, F. Hollow Core-shell Structured Si@Nial-LDH Composite as High-performance Anode Material in Lithium-ion Batteries *Electrochim. Acta* **2020**, *331*, 135331.

(S9) Liu, Z.; Zhao, Y.; He, R.; Luo, W.; Meng, J.; Yu, Q.; Zhao, D.; Zhou, L.; Mai, L. Yolk@Shell SiO_x/C Microspheres with Semi-graphitic Carbon Coating on the Exterior and Interior Surfaces for Durable Lithium Storage *Energy Storage Mater.* **2019**, *19*, 299−305.

(S10) He, Y.; Han, F.; Wang, F.; Tao, J.; Wu, H.; Zhang, F.; Liu, J. Optimal Microstructural Design of Pitch-derived Soft Carbon Shell in Yolk-shell Silicon/Carbon Composite for Superior Lithium Storage *Electrochim. Acta* **2021**, *373*, 137924.

(S11) Hu, L.; Luo, B.; Wu, C.; Hu, P.; Wang, L.; Zhang, H. Yolk-shell Si/C Composites with Multiple Si Nanoparticles Encapsulated into Double Carbon Shells as Lithium-ion Battery Anodes *J. Energy Chem.* **2019**, *32*, 124−130.

(S12) Yan, J.; Gao, C.; Qi, S.; Jiang, Z.; Jensen, L. R.; Zhan, H.; Zhang, Y.; Yue, Y. Encapsulation of Nano-Si into MOF Glass to Enhance Lithium-ion Battery Anode Performances *Nano Energy* **2022**, *103*, 107779.

(S13) Kwon, H.-T.; Park, A.-R.; Lee, S.-S.; Cho, H.; Jung, H.; Park, C.-M. Nanostructured Si-$FeSi_2$-graphite-C Composite: An Optimized and Practical Solution for Si-based Anodes for Superior Li-ion Batteries *J. Electrochem. Soc.* **2019**, *166*, A2221.

(S14) Wang, Y.-Q.; Yang, X.-X.; Ren, M.-X.; Lei, B.-Y.; Hou, Y.-L.; Meng, W.-J.; Zhao, D.-L. 3D CNTs Networks Enable Core-shell Structured Si@Ni Nanoparticle Anodes with Enhanced Reversible Capacity and Cyclic Performance for Lithium Ion Batteries *Int. J. Hydrogen Energy* **2021**, *46*, 16179−16187.

(S15) An, W.; Gao, B.; Mei, S.; Xiang, B.; Fu, J.; Wang, L.; Zhang, Q.; Chu, P. K.; Huo, K. Scalable Synthesis of Ant-nest-like Bulk Porous Silicon for High-performance Lithium-ion Battery Anodes Nat. Commun. **2019**, *10*, 1447.

(S16) Dong, Z.; Du, W.; Yan, C.; Zhang, C.; Chen, G.; Chen, J.; Sun, W.; Jiang, Y.; Liu, Y.; Gao, M.; Gan, J.; Yang, Y.; Pan, H. A Novel Tin-bonded Silicon Anode for Lithium-ion Batteries *ACS Appl. Mater. Interfaces* **2021**, *13*, 45578−45588.

(S17) Nava, G.; Schwan, J.; Boebinger, M. G.; McDowell, M. T.; Mangolini, L. Silicon-core−carbon-shell Nanoparticles for Lithium-ion Batteries: Rational Comparison Between Amorphous and Graphitic Carbon Coatings *Nano Lett.* **2019**, *19*, 7236−7245.

(S18) Bi, X.; Tang, T.; Shi, X.; Ge, X.; Wu, W.; Zhang, Z.; Wang, J. One-step Synthesis of Multi-core-void@shell Structured Silicon Anode for High-performance Lithium-ion Batteries *Small* **2022**, *18*, 2200796.

(S19) Li, J.; Wang, T.; Wang, Y.; Xu, Z.; Mateen, A.; Yan, W.; Li, H.; Mujear, A.; Chen, J.; Deng, S.; Gao, G.; Zheng, C.; Zhu, Y.; Di, Z.; Mei, Y.; Bao, Z. Solid-liquid-solid Growth of Doped Silicon Nanowires for High-performance Lithium-ion Battery Anode *Nano Energy* **2025**, *133*, 110455.

(S20) Tian, H.; Tian, H.; Yang, W.; Zhang, F.; Yang, W.; Zhang, Q.; Wang, Y.; Liu, J.; Silva, S. R. P.; Liu, H.; Wang, G. Stable Hollow-structured Silicon Suboxide-based Anodes toward High-performance Lithium-ion Batteries *Adv. Funct. Mater.* **2021**, *31*, 2101796.

(S21) Zhang, M.; Bai, N.; Lin, W.; Wang, H.; Li, J.; Ma, L.; Wang, X.; Zhang, D.; Cao, Z. Carbon-encapsulated Silicon Ordered Nanofiber Membranes as High-performance Anode Material for Lithium-ion Batteries *J. Alloys Compd.* **2025**, *1010*, 177012.
